# Could Cultured Meat Be a Sustainable and Safe Source of Protein?

**DOI:** 10.1002/mnfr.70319

**Published:** 2025-11-20

**Authors:** Denise Mafra, Liana Trugilho, Fabiana Nerbass, Peter Stenvinkel, Ludmila F. M. F. Cardozo

**Affiliations:** ^1^ Graduate Program in Medical Sciences Fluminense Federal University (UFF) Niterói Rio de Janeiro Brazil; ^2^ Graduate Program in Biological Sciences – Physiology Federal University of Rio de Janeiro (UFRJ) Rio de Janeiro Brazil; ^3^ Graduate Program in Nutrition Sciences Fluminense Federal University (UFF) Niterói Rio de Janeiro Brazil; ^4^ Research Department Fundação Pró‐Rim Joinville Santa Catarina Brazil; ^5^ Division of Renal Medicine Department of Clinical Science Technology and Intervention Karolinska Institutet Stockholm Sweden; ^6^ Graduate Program in Cardiovascular Sciences Fluminense Federal University (UFF) Niterói Rio de Janeiro Brazil

**Keywords:** cultured meat, environment, greenhouse gas emissions, health, red meat

## Abstract

Red and processed meat includes high‐quality proteins and essential sources of micronutrients, such as iron, zinc, and vitamin B12; however, high consumption is linked to an increased risk of chronic disease burden and also harms environmental sustainability, as methane produced by ruminant animals is a significant contributor to greenhouse gas emissions. New strategies to mitigate chronic disease risk and methane production have been developed, and the replacement of natural beef with “cultured beef” has been discussed. Cultured Meat is an innovative field that addresses human nutrition and environmental preservation. However, further research is needed regarding the effects on human health, including the chronic burden of lifestyle‐related diseases. This mini‐review summarizes recent findings on the production technologies, environmental footprint, and nutritional composition of cultured meat, highlighting both its promises and current limitations. Notably, no clinical trials have evaluated its health effects in humans, and sustainability claims remain largely theoretical and dependent on renewable energy sources.

## Graphical abstract

Cultured meat could be a sustainable alternative to traditional beef, providing potential benefits for both human health and environmental preservation.

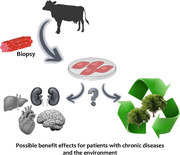

**Abbreviations**
GHG: greenhouse gas
PUFAs: polyunsaturated fatty acids
CH_4_
: methane
N_2_O: nitrous oxide

## 1 Introduction

Red meat (beef, lamb, and pork) is an important source of essential amino acids and micronutrients, including iron (heme), zinc, and vitamin B_12_. However, emerging scientific evidence suggests that increased consumption of red and processed meat is associated with health problems, including chronic diseases [1]. Moreover, increased consumption of red meat is also associated with greenhouse gas emissions, including carbon dioxide and methane. Microbial fermentation in cattle and sheep represents a high percentage of emissions (64% is methane produced by Archaea, a domain of microorganisms different from bacteria) [2]. The discussion surrounding food production has focused on enhancing the health and quality of life for the general population and patients with chronic illnesses, while also promoting environmental sustainability. Cultured meat is a novel product that may be a healthier protein option and a good strategy for reducing greenhouse gas emissions. However, no study has investigated the effects of cultured meat intake on human health, even in patients with chronic diseases. Although popularization is growing, there are still many discussions on the acceptance, organoleptic properties, nutritional properties, and religious status [3].

Given the growing interest in alternatives to conventional meat, this narrative review aims to investigate whether cultured meat can be a viable and safe source of dietary protein for human health and a sustainable solution to environmental challenges. To address this, key parameters of sustainability were evaluated, including greenhouse gas emissions**, **land and water use, and resource efficiency, as well as parameters of safety and nutritional adequacy, such as protein and micronutrient composition**, **presence of bioactive compounds, and potential health effects. Additionally, consumer acceptance and technological limitations that may influence the adoption of cultured meat were considered. This review aims to contribute to the scientific discussion by identifying current evidence, research gaps, and future directions for integrating cultured meat into sustainable and healthy food systems for patients with chronic diseases.

## 2 Method

This narrative review was conducted to synthesize the most recent and relevant scientific literature on cultured meat and its potential implications for human health and environmental sustainability. A comprehensive literature search was conducted using the electronic databases PubMed**, **Scopus, and Web of Science, covering the period from January 2021 to July 2025. The search strategy included combinations of the following keywords and MeSH terms: “cultured meat,” “lab‐grown meat,” “in vitro meat,” “cell‐based meat,” “sustainability,” “greenhouse gas emissions,” “chronic disease,” “nutritional value,” and “consumer acceptance,” Boolean operators (AND, OR) were applied to optimize the sensitivity and specificity of the searches.

Articles were included if they met the following criteria: (1) original research, reviews, or perspectives published in peer‐reviewed journals; (2) studies addressing cultured meat in the context of nutrition, environmental impact, safety, or consumer perceptions; and (3) studies published in English. Exclusion criteria included articles not focused on cultured meat (e.g., plant‐based meat only), studies without accessible full texts, or publications in non‐scientific journals.

### 2.1 Red Meat

Meat consumption has increased worldwide, driven by population growth, societal changes, domestication, and technological advancements [3]. Although there are differences between countries, high‐income nations tend to have higher levels of meat consumption, often surpassing nutritional requirements [4]. This is influenced by factors beyond diet alone, including economic and social considerations [3].

Red meat contains muscle fibers and other tissues, including connective, adipose, vascular, and nervous tissues [5]. It is a significant source of essential amino acids [6], including taurine, creatine, carnosine, anserine, and 4‐hydroxyproline, which provide energy and support immunological and oxidative defenses [7]. Meat is also a source of lipids, including saturated (palmitic, stearic, and myristic acids) and polyunsaturated fatty acids (PUFAs; linoleic acid [n‐6] and α‐linolenic acids [n‐3]) [8]. Meat also provides vitamins and minerals, such as cobalamin (B12) and zinc [6]. Furthermore, meat is the primary source of heme‐iron [9], has high bioavailability, and contributes to the prevention of anemia [10].

However, high consumption of red meat leads to a high intake of saturated fats. It is associated with inflammatory responses and an increased risk for cardiovascular disease, raising concerns about increasing meat consumption due to its fatty acid content [11]. Processed meat has undergone steps to improve its durability or organoleptic properties, such as salting, curing, fermentation, smoking, or adding preservatives, as in ham, sausages, salami, bacon, and so forth [12], which offer to the consumer a food rich in salt, saturated fat, nitrates and nitrites, which produce N‐nitroso compounds [12]. Meta‐analyses have demonstrated a higher risk of several cancer types [13], hypertension, stroke, and incident cardiovascular disease [14] associated with high red and processed meat consumption. Dietary patterns that include a high intake of these food groups also increase the risk of type 2 diabetes [15]. Novel animal studies data link cancer risk with red meat consumption in a carnivorous diet [16].

In addition to health concerns, meat consumption raises critical environmental issues [17]. Ruminants, the leading livestock source of greenhouse gas (GHG), contribute directly and indirectly to climate change and environmental impacts [18], promoting the emission of methane (CH_4_) and nitrous oxide (N_2_O), not only from manure management but also due to enteric fermentation, which is responsible for about 40% of GHG emissions in agriculture [19]. Additionally, since manure is commonly used in fertilization, it contains antibiotics administered to animals, thereby contributing to environmental antibiotic resistance [20]. Likewise, fertilizer application, deforestation, and land use for livestock feed also contribute to N_2_O and carbon dioxide emissions [19, 21]. GHG emissions are associated with pulmonary harm due to gas emissions and noxious particulates, such as endotoxin, affecting non‐farming populations [22]. Another critical negative impact of livestock and meat production is the use of surface water and groundwater, also known as the blue water footprint, which involves a significant amount of water being used for grain crops to feed the animals and for direct consumption [23]. Deforestation and polluted soil with reduced biodiversity [24] may indirectly contribute to adverse changes in human health. However, as meat is still a vital food in economic, nutritional, and social aspects, it is crucial to find solutions that address the appeal of red meat in society.

Conventional meat production raises concerns regarding sanitation, ethics, and the environment due to its high‐water usage, land demand, and links to deforestation and greenhouse gas emissions [19]. The SARS‐CoV‐2 virus and the COVID‐19 crisis raised new concerns over intensive livestock farming and the risk of new pandemic zoonoses [25, 26, 27]. In a review, researchers reported that alternative proteins, including plant‐based proteins, single‐cell proteins, insect proteins, duckweed, and cultured meat, can minimize the impact on resource use by approximately 70% compared to traditional protein sources [28]. Given the sanitary, environmental, and moral risks associated with conventional meat production, interest in cultured meat has been growing [26, 29].

### 2.2 Cultured Meat

Information about bioartificial muscles is not new, as a 1932 book by Churchill, titled “Thoughts and Adventures,” mentions this term [30]. In the late 1990s, Willem van Eelen registered the first patent for a method of meat culture production [31]. After that, two projects were undertaken in the early 2000s to cultivate tissue for food: one funded by NASA and the other by a team at the Tissue Project Culture and Art [32, 33]. However, the most notable work came in 2013 from Maastricht University, led by Mark Post. After two years of development, he produced the world's first cultured beef burger, which he prepared and enjoyed on live television. The price of this ground‐breaking work was over $300 000 in 2013 [34, 35]. After 2013, several companies entered the cultured meat industry, with various products in development, including chicken, beef, pork, and seafood. Many companies have been established worldwide since the end of 2020, with an interest in cultured meat end products, raw materials, or equipment used in production [36, 37]. A preliminary study of the production cycle of cultured meat showed possible benefits from its consumption. Compared to traditional meat production, it could use 7%–45% less energy, 99% less land, and 96% less water and generate 78%–96% less GHG emissions [38].

Cultured meat was initially referred to as “in vitro meat,” in which cells and tissues are cultured *in a laboratory setting*. It is also called clean, cell‐based, laboratory‐grown, or cultured meat [39, 40]. Culturally cultured meat production aims to replicate meat produced conventionally, but “not in a cow,” instead using stem cells and tissue culture [35, 39]. Several technologies are employed for in vitro meat production, with some being more commercially oriented and others more viable on a laboratory scale [41]. There are two primary classifications of methods used to produce cultured meat. The first is the self‐organizing technique, based on a biopsy or explant from the donor animal. The second technique is scaffolding‐based, which multiplies embryonic myoblasts or adult skeletal muscle cells [42]. The scaffold‐based technique involves a fixed substrate or scaffold composed of collagen or carrier microspheres within the bioreactor's growth medium. In this way, myofibers are produced that can be harvested, processed, and consumed as meat products [41]. It is essential to note that the culture medium containing recombinant proteins or fetal bovine serum is necessary to enhance muscle cell proliferation, which is contradictory to the discussion of environmental damage. New research has contributed to increasing the production process of cultured meat, such as a culture system of muscle cells using serum‐free alginate microcapsules [43], the use of methyl‐β‐cyclodextrin (MβCD) to inhibit differentiation of myoblasts, but maintaining the proliferation [44], or using an oleogel system as a fat substitute [45].

The protein content and the composition of cells in culture compared to that of traditional red meat have yet to be established [46]. The composition of fatty acids influences caloric density and can vary with the presence of saturated, unsaturated, and trans‐unsaturated fats. Fatty acids can be added by co‐culturing adipocytes derived from adipose stem cells, synthesizing various saturated and unsaturated fatty acids [47].

Meat is also a significant source of essential minerals, including iron, zinc, and selenium. In muscle tissue, iron is present as part of a heme group in myoglobin or stored in a complex with ferritin in a non‐heme form [48]. The consumption of iron in the heme form is essential because it is more easily absorbed [49]. In basal cell culture media, other minerals, such as zinc and selenium, are absent (or in low concentration) [46]. As meat is an essential source of B vitamins, such as B_12_ [50], cultured meat must also contain these vitamins as a substitute for traditional meat. However, vitamins are included in the medium for optimal cell proliferation [51]. It remains to be determined whether this results in adequate vitamin levels in cultured meat compared to traditional meat [46].

**FIGURE 1:**
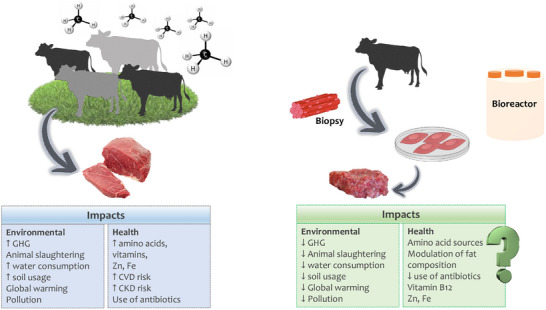
The possible impact of cultured meat intake on the environment and health. Red meat production has a significant impact on the environment, with high greenhouse gas emissions (GHG). It can also increase the risk of chronic diseases. The emerging technology with cultured meat production may contribute to a sustainable diet, but the effects on human health are still challenging. Image created with Biorender.com.

Recent literature has critically assessed the environmental sustainability and safety aspects of cultured meat. Tavan et al. highlighted that the claims regarding the environmental benefits of cultured meat are often overly optimistic and lack robust scientific validation. Although it is frequently promoted as a solution to reduce greenhouse gas emissions, land use, and water consumption, life cycle assessments have shown that cultured meat production remains highly energy‐intensive. Unless this energy demand is met by renewable sources, the net environmental gain may be negligible or even negative [52]. Regarding food safety, although cultured meat has potential advantages, including reduced exposure to antibiotics, hormones, and zoonotic pathogens, there is still a lack of long‐term studies assessing its effects on human health. The production process, which includes the use of scaffolds, growth media, and bioreactors, introduces new variables and potential risks that are not yet fully understood. To date, no clinical trials in humans have been conducted, and the nutritional profile of cultured meat remains speculative. Figure 1 provides a visual summary of how cultured meat currently performs across different sustainability and safety criteria. It illustrates that while cultured meat may outperform conventional meat in certain aspects, such as the absence of animal slaughter and potential pathogen control, it still falls short in others, particularly in terms of energy use and evidence‐based safety validation.

Recently, a discussion has arisen regarding the need to simplify the cultured meat production process, increase scalability, and also make cultured meat less artificial and more affordable [53, 54, 55]. In this direction, Wang et al. [54] used proanthocyanidins and chitosan dialdehyde as double cross‐linkers with collagen, aiming to prepare a hybrid 3D edible structure in the production of cultured meat. The authors reported that this cultured meat, when cooked, presented colors and flavors with characteristics similar to beef, excluding the use of artificial dyes. The use of probiotic bacteria has also been suggested in cultured meat production. The introduction of bacteria into cultured meat production can play a role at various stages, allowing for the co‐culturing of mammalian cells and bacteria. This approach can promote greater scalability and nutrient production, as well as enhanced cell support, improved contamination control, and better texture and flavor, among other potential benefits [55].

Consumer acceptance of cultured meat has been widely studied in recent years [56]. A crucial point is whether consumers will purchase cultured meat, as consumer acceptance is necessary for trade and delivery of long‐term benefits to society [39]. The acceptance of cultured meat is reduced due to concerns over safety, wholesomeness, flavor, texture, price, and the perceived lack of naturalness, despite knowledge of potential environmental and animal welfare benefits [56].

It has been shown that consumers tend to reject the name “in vitro meat,” with the term “cultured” being less unappetizing than the terms “artificial” and “laboratory‐grown” [57]. Knowledge of technology can enhance acceptance, and food neophobia can contribute to the rejection of cultured meat [58]. The acceptance of new food, such as cultured meat, depends on the level of information on it, whether pre‐existing or provided [59]. Furthermore, Etter et al. [60] demonstrated the challenge of convincing consumers to accept cultured meat (cultured beef, cultured pork, cultured chicken) as alternatives to conventional meat, as consumers tend to favor familiar and plant‐based protein sources over other ingredients in meat alternatives. The preference for authenticity represents a key barrier to the widespread adoption of all types of cultured meat [60]. Table 1 summarizes the main recent results found in the literature regarding cultivated meat and consumer acceptance.

**TABLE 1:** Studies involving cultivated meat and consumer acceptance.

References	Sample	Methods	Results
Mendes et al. [61]	809 Brazilian participants, 345 men and 464 women over 18 years	Online questionnaire with multiple‐choice and open‐ended questions	38.3%—had heard about cultured meat; 32.8%—reported interest in consuming cultured meat, 26.0%—would not consume it due to neophobia, artificiality, lack of knowledge; and not being healthy. 41.2%—didn't know
Pareti et al. [62]	59 Chinese participants, including 32 males and 27 females, 20–60 years	Qualitative research with focal groups, recruitment conducted on social media platforms	Low level of understanding about meat grown from animal cells; Questions about artificial flavor, high price, market monopolization, and negative effects on traditional animal farming and job losses.
Stubelj et al. [63]	458 Slovenian participants, 194 men and 264 women, 18–83 years	Cross‐sectional study with closed questionnaire for data collection through Facebook groups and email invitations	21% were open to consume cultured meat; Women were less inclined to try cultured meat.
Gençer Bingöl et al. [64]	504 young adults (18–40 years), 176 men and 328 female, mean age: 24.4	Cross sectional study through online questionnaire form via phone and email	61.1% had prior knowledge of cultured meat; 33.5% would accept consuming it; Acceptance decreased with increasing age; Acceptance increased with higher education.
Khaleel et al. [65]	577 United Arab Emirates participants, 167 males and 410 females, over 18 years.	A cross‐sectional study using an online questionnaire with closed questions.	59.3%—unfamiliar with the name “cultured meat”; 35%—would try cultured meat, 69%—no interest in replacing conventional meat with cultured meat
Chriki et al. [66]	1025 Arab participants	Online survey	40%—would try artificial meat; 36%—aversion, 22.7%—unsure; 55%—would pay less for artificial meat compared to conventional meat.
Sikora et al. [67]	1553 Poles participants, 1217 females, 329 males and 7 others; over 18 years.	Cross‐sectional online questionnaire	63%—familiar with the concept of cultured meat 54%—would buy it when it became available. The motivations for acceptance were: 76% to reduce animal suffering, 67% environmental impacts, and 58% curiosity.
Pilařová et al. [68]	740 respondents, 301 Generation Z (1997–2012) and 439 Generation Y (1981–1996)	Online questionnaire on social networks: Facebook, LinkedIn and Twitter.	Generation Z considers cultured meat healthier than conventional meat compared to Generation Y; Generation Z is less concerned about the effects of cultured meat on human health.
Vural et al. [69]	151 meat eaters and 44 non‐meat eaters, from UK, over 18 years	Online survey	Meat consumers judged cultured meat products as equally or healthier, but more disgusting when compared to conventional meat products.
Dupont J et al. [70]	497 German participants; 249 male and 248 female; over 18 years	Online questionnaire	32.2% had heard of cultured meat and were familiar with its meaning; 30.2% had never heard of cultured meat; 58.4% expressed a favorable opinion of consuming cultured meat burgers.
Hamlin et al. [71]	254 New Zealanders from University of Otago; over 18 years	Online surveys were done “face‐to‐face”	33.86% reported being unaware of clean meat 59.06% had already heard about it Cultured meat—still presented disadvantages in terms of purchase intentions when compared to plant‐based or animal protein.
Bogueva and Marinova [72]	478 Australian Generation Z adults, 238 male and 240 female; born between 1995 and 2003, aged 18 to 26.	A questionnaire, containing multiple‐choice and open‐ended questions	28% of participants accepted cultured meat as a new protein source; 72% who reported not accepting cultured meat described it as “unnatural,” “abnormal,” “artificial,” and “abnormal.” 17% of those who reject it perceive cultured meat as “chemically produced,” “highly processed,” “Frankenstein food,” and “not what our generation needs.”
de Oliveira et al. [73]	225 Brazilian participants; 46.7% male and 53.3 female; over 18 years	Online survey	80.9% of participants would try it, 61.3% would eat it regularly, and 56.9% would substitute it for conventional red meat.
Szejda et al. [74]	2292 participants from the US and 2270 from the UK; aged 18–74	Online survey	80%—accept cultured meat, with 40% reasonably or moderately likely to try it, and 40% highly likely to try it;

Considering a hypothetical future (when production and consumer barriers have been overcome) and cultured meat becomes available at an affordable price, the nutritional effects of cultured meat consumption require further study. Additionally, it is crucial to determine if we can modulate the composition of amino acids. The fat composition should also be modulated for specific diseases, with a reduction in saturated fatty acids and an increase in polyunsaturated fatty acids. This would improve an altered blood lipid profile and reduce cardiovascular risk [75].

Cultured meat should preserve or improve the heme‐iron amount and provide a reasonable oral iron intake for patients with chronic diseases. Zinc and vitamin B12 levels should also be monitored when cultured meat is tested. Compared to “natural” red meat, cultured meat will still be an animal‐sourced protein, which can increase acid production and lead to gut dysbiosis. Additionally, microbiological safety and gut health remain underexplored areas in the development of cultured meat. Most of the key microbial contaminants found in conventional meat are also expected to pose risks for cultivated meat, potentially impacting its stability and safety [76]. However, there is currently no scientific evidence on how microbial contaminants specifically affect either the biotechnological production process or the final product. Therefore, it is crucial to implement strict hygienic manufacturing protocols and standardized cell culture practices. Furthermore, clear guidance on preparation, handling, cooking temperatures, and storage should be included on product labels. Since the matrix is new, potential interactions with the human gut microbiota should also be investigated in future studies [77].

It is essential to note that cultured meat must be well accepted by the population in terms of its organoleptic characteristics, including flavor, aroma, and texture, without the use of additives. Otherwise, it can become just as harmful as processed meat.

### 2.3 Future Perspectives

Most published studies on cultured meat have focused on consumer perceptions, while data on the effects of cultured meat intake on human metabolism and health remain limited. Nonetheless, cultured meat has the potential to become a viable alternative to traditional animal protein sources in the future. Although the long‐term implications are still unclear, this emerging field raises essential questions: Is cultured meat safe? Could it reduce reliance on conventional animal farming and mitigate environmental degradation? Can it contribute to pandemic prevention by decreasing zoonotic transmission?

However, its long‐term health safety, nutritional composition, scalability, and social acceptance still require full scientific validation.

Future research should focus on several critical areas to advance the understanding and adoption of cultured meat. First, technological innovations are needed to improve the efficiency, scalability, and cost‐effectiveness of production, including the development of serum‐free culture media, sustainable scaffolding materials, and energy‐efficient bioreactors. Second, experimental studies and clinical trials should be conducted to assess the health impacts of cultured meat consumption, particularly in populations at risk for chronic diseases.

Third, consumer behavior research should explore sensory, cultural, and psychological barriers to acceptance and inform public education and communication strategies. Studies conducted in several countries have revealed low to moderate levels of consumer familiarity and willingness to consume cultured meat, with key barriers including food neophobia, perceived artificiality, and a lack of trust in the product. These findings underscore the need for targeted educational campaigns, transparent labeling, and enhanced sensory characteristics to foster acceptance.

Fourth, comparative life cycle assessments using real‐world energy sources are crucial for verifying the environmental benefits of cultured meat compared to traditional livestock farming. Ultimately, interdisciplinary collaboration among nutritional science, food technology, environmental science, and policy will be crucial to the development of safe, nutritious, and widely accepted cultured meat products, supported by robust regulatory frameworks.

While the promise of cultured meat is compelling, its successful integration into the global food system depends on overcoming the scientific, technological, and societal challenges it presents. This review lays the groundwork for future research that aims to align food innovation with public health and environmental sustainability. Despite increasing worldwide interest in cultured meat as a sustainable alternative to traditional animal‐based proteins, there is a notable lack of peer‐reviewed scientific data on its nutritional and biochemical properties. Most studies concentrate on technological feasibility, production costs, and consumer acceptance, while objective analyses of the nutritional profile, including amino acid composition, micronutrient levels, and bioavailability, remain limited. This gap presents a significant obstacle to establishing cultured meat as a safe and nutritious option. Our review does not intend to offer definitive comparisons but to highlight the scarcity of solid evidence and stress the urgent need for systematic research in this field. As global conversations about food sustainability continue to grow, prioritizing high‐quality nutritional research on cultured meat should be a key goal for both academic institutions and regulatory bodies.

## Funding

Conselho Nacional de Pesquisa (CNPq), Coordenação de Aperfeiçoamento de Pessoal de Nível Superior (CAPES) and Fundação Carlos Chagas Filho de Amparo à Pesquisa do Estado do Rio de Janeiro (FAPERJ) support Denise Mafra research. Fundação de Amparo à Pesquisa do Estado do Rio de Janeiro (FAPERJ) support Ludmila FMF Cardozo.

## Conflicts of Interest

The authors declare no conflicts of interest.

## Data Availability

Data sharing not applicable to this article as no datasets were generated or analyzed during the current study.
